# Limited Effectiveness of a Skills and Drills Intervention to Improve Emergency Obstetric and Newborn Care in Karnataka, India: A Proof-of-Concept Study

**DOI:** 10.9745/GHSP-D-16-00143

**Published:** 2016-12-23

**Authors:** Beena Varghese, Jayanna Krishnamurthy, Blaze Correia, Ruchika Panigrahi, Maryann Washington, Vinotha Ponnuswamy, Prem Mony

**Affiliations:** a Public Health Foundation of India, Gurugram, India.; b Karnataka Health Promotion Trust, Bangalore, India.; c St. Johns Research Institute, Bangalore, India.

## Abstract

**Objective:**

The majority of the maternal and perinatal deaths are preventable through improved emergency obstetric and newborn care at facilities. However, the quality of such care in India has significant gaps in terms of provider skills and in their preparedness to handle emergencies. We tested the feasibility, acceptability, and effectiveness of a “skills and drills” intervention, implemented between July 2013 and September 2014, to improve emergency obstetric and newborn care in the state of Karnataka, India.

**Methods:**

Emergency drills through role play, conducted every 2 months, combined with supportive supervision and a 2-day skills refresher session were delivered across 4 sub-district, secondary-level government facilities by an external team of obstetric and pediatric specialists and nurses. We evaluated the intervention through a quasi-experimental design with 4 intervention and 4 comparison facilities, using delivery case sheet reviews, pre- and post-knowledge tests among providers, objective structured clinical examinations (OSCEs), and qualitative in-depth interviews. Primary outcomes consisted of improved diagnosis and management of selected maternal and newborn complications (postpartum hemorrhage, pregnancy-induced hypertension, and birth asphyxia). Secondary outcomes included knowledge and skill levels of providers and acceptability and feasibility of the intervention.

**Results:**

Knowledge scores among providers improved significantly in the intervention facilities; in obstetrics, average scores between the pre- and post-test increased from 49% to 57% (*P*=.006) and in newborn care, scores increased from 48% to 56% (*P*=.03). Knowledge scores in the comparison facilities were similar but did not improve significantly over time. Skill levels were significantly higher among providers in intervention facilities than comparison facilities (mean objective structured clinical examination scores for obstetric skills: 55% vs. 46%, respectively; for newborn skills: 58% vs. 48%, respectively; *P*<.001 for both obstetric and newborn), along with their confidence in managing complications. However, this did not result in significant differences in correct diagnosis and management of complications between intervention and comparison facilities. Shortage of trained nurses and doctors along with unavailability of a consistent supply chain was cited by most providers as major health systems barriers affecting provision of care.

**Conclusions:**

Improvements in knowledge, skills, and confidence levels of providers as a result of the skills and drills intervention was not sufficient to translate into improved diagnosis and management of maternal and newborn complications. System-level changes including adequate in-service training may also be necessary to improve maternal and newborn outcomes.

## Abstract (precis)

Skills refresher training combined with emergency drills improved knowledge, skills, and confidence of providers but was not sufficient to improve diagnosis and management of maternal and newborn complications. Systems-level changes, including consistent availability of equipment and supplies, adequate human resource staffing, and supportive supervision, are likely needed to improve maternal and newborn outcomes.

## BACKGROUND

Over the last decade, the maternal mortality ratio in India has declined by about 35%, from 254 per 100,000 live births in 2005–2007 to 167 in 2011–2013), and the neonatal mortality rate has declined by 28%, from 39 per 1,000 live births in 2005 to 28 in 2015.^1^ Levels vary widely across states within the country.^2^^,^^3^

To achieve further declines, both central and state governments have been shifting their focus toward improving the quality of institutional delivery care. The Seventh Common Review Mission of the National Rural Health Mission reported that, although there has been improvements in the labor room conditions and in the availability of essential drugs and supplies, the status of emergency obstetric and newborn care needs further attention in many states, especially in terms of providers' skill levels in treating maternal and newborn complications.^4^ A facility assessment conducted during 2010 in Karnataka, a southern state in India, highlighted that non-availability of competent staff and of supplies were the major gaps in emergency obstetric care provision. The assessment recommended improving clinical skills competencies and facility preparedness, especially emergency preparedness through a continuous support mechanism.^5^ Recent studies on emergency preparedness have reported that emergency obstetric drills have resulted in improvements in the knowledge and skill levels of providers as well as in facility preparedness.^6^^–^^9^

Recent studies have reported improved provider knowledge and skills as a result of emergency obstetric drills.

It was thus envisaged that a “skills and drills” intervention—that is, combining refresher training with a series of emergency drills—would help improve the skill levels of providers and strengthen system preparedness to handle obstetric and newborn emergencies. The goal of this proof-of-concept study was to test the feasibility and effectiveness of a skills and drills intervention. The specific objectives were to:

1. Understand acceptability and feasibility of the skills and drills intervention at public facilities in Karnataka, India.
2. Assess the effectiveness of the skills and drills intervention to improve the diagnosis and management of selected maternal and newborn complications consisting of pregnancy-induced hypertension (PIH), postpartum hemorrhage (PPH), and birth asphyxia.

## METHODOLOGY

### Study Setting and Design

The study was conducted in 2 northern districts (Bagallkot and Koppal) of Karnataka, India. Among the southern states of India, Karnataka reports the highest maternal mortality ratio, at 133 per 100,000 live births (2012–2013), and the second highest infant mortality rate, at 31 per 1,000 live births (2013).^2^^,^^3^ Within Karnataka, the 8 northern districts are economically disadvantaged and have significant disparities in health infrastructure and service delivery compared with the rest of the state.^10^^,^^11^

We used a quasi-experimental design with 4 intervention and 4 comparison facilities within these 2 districts over a 14-month time period (July 2013 to September 2014) to test the effectiveness of the skills and drills intervention. The facilities were sub-district-level government health facilities with basic emergency obstetric and newborn capabilities, with 2 facilities equipped to handle cesarean deliveries. The intervention and comparison facilities were comparable in terms of delivery load, type of deliveries, infrastructure, and human resource capacity (Table 1). We hypothesized that the planned intervention would improve the skill and knowledge levels of providers, improve emergency preparedness, and strengthen team work, resulting in improved diagnosis and management of selected maternal and newborn complications at the intervention facilities.

**TABLE 1.** Characteristics of Intervention and Comparison Facilities, Selected Districts of Karnataka, India, July 2013 to September 2014

Parameters	Intervention (n = 4)	Comparison (n = 4)
Type of facility		
Sub-district hospital	3	3
Community health center	1	1
Human resource capacity		
Obstetrician/trained in EmOC	6	3
Pediatrician	0	3
Physician	2	2
Anesthetist	1	1
Nurses	64	68
No. of deliveries per quarter, 2013, mean	1,840	1,680
No. of cesarean deliveries per quarter, 2013, mean	119	98

Abbreviation: EmOC, emergency obstetric care.

### Intervention

The intervention (at the 4 intervention facilities) included a skills refresher session and emergency drills held every 2 months combined with supportive supervision provided by an external team of trainers who were obstetric and pediatric specialists and nurses. This external team consisted of 10 obstetrics specialists and 10 pediatric specialists along with 8 nurses selected from medical colleges across 3 cities or towns of Karnataka (Bangalore and Mysore cities and Bagalkot town). Each of the selected specialists had more than 10 years of clinical experience in their respective fields and most of them were professors or associate professors at medical colleges; the obstetric and pediatric nurses had more than 5 years of experience in their respective fields and had some public health experience, either serving at public facilities or working in the past with research teams. They were all apprised of the travel requirement to study facilities, which required an overnight journey for all except for those from Bagalkot. The team of trainers received an orientation over 2.5 days prior to the intervention; the orientation covered disease management protocols, skill stations demonstrations, and drill exercises. The Institute for Clinical Effectiveness and Health Policy, Argentina, provided the training on the emergency drill exercises.

We assessed effectiveness of an intervention combining a skills refresher session, emergency drills, and supportive supervision.

The intervention activities consisted of:

1. **Clinical skills refresher session:** The skills refresher training session (conducted by the external team of obstetricians and pediatricians) was a one-time activity held over 2 days at the 4 intervention facilities. The session focused on technical skills and competence building through didactic sessions and skill stations, covering topics related to physical examination of a woman in labor, active management of third stage of labor (AMTSL), emergency preparedness for complications, immediate newborn care, warm chain and feeding of the newborn, neonatal resuscitation, and supportive care for a sick newborn. Participants included all nurses in the labor rooms as well as medical doctors and specialists (if any were available at these facilities).
2. **Emergency drills:** Once every 2 months, emergency drills for one obstetric complication (either PPH or eclampsia) and one neonatal complication (birth asphyxia) were simulated using a prewritten script; the drills were conducted by a team of 3 trainers (from the external team) comprising an obstetric or pediatric specialist and 2 nurses. The specialist facilitated the drill exercise by providing clinical information relevant to the enacted case scenario while one of the nurses acted as the patient and the other nurse as the patient's relative; health facility staff members were instructed to respond to the simulated emergency situation as close to the real-life local context as possible. The facilities did not receive any prior notice of when the drills would be conducted. The simulation exercises related to birth asphyxia used a NeoNatalie simulator from Laerdal—an inflatable simulator designed to teach basic neonatal resuscitation skills. Each drill, lasting about 45 minutes, was videotaped and played back to the facility staff. The trainers conducted a detailed debriefing to identify both health systems issues (such as availability of drugs and supplies) and gaps in skills and team preparedness (such as communication or team rapport).
3. **Supportive supervision:** While the team of trainers conducted the drills, a second team of trainers provided supportive supervision to the facility staff on duty by observing practices such as deliveries, newborn feeding, infection control, and availability of critical drugs and supplies, and reviewing delivery and complication case sheets. Based on observations made during the drills and the supportive supervision, the trainer team then debriefed the facility staff and developed a joint action plan for the next cycle. Each subsequent visit started with a review of the action plan and gaps identified during the previous visit.

### Outcome Measures

The primary outcome measure was the timely and correct diagnosis and management of direct obstetric complications (PPH and pre-eclampsia/eclampsia) and the newborn complication of birth asphyxia. PPH was defined as the loss of 500 mL or more of blood during or within 24 hours of childbirth^12^; pre-eclampsia was defined as systolic blood pressure ≥140 mm Hg or diastolic blood pressure ≥90 mm Hg with proteinuria after 20 weeks gestation in women with a previously normal blood pressure; eclampsia was defined as the presence of convulsions with signs of pre-eclampsia^12^; and birth asphyxia was defined as the failure to establish breathing at birth or not crying at birth.^13^

Secondary outcomes included knowledge and skill levels of the providers related to maternal and newborn care. For obstetrics, we focused on assessment of labor and initial management of hypertensive disorders of pregnancy and postpartum hemorrhage; for newborn care, we focused on infection control, neonatal resuscitation, and care of low birthweight babies. In addition, we measured the acceptability and feasibility of the intervention as perceived by facility providers.

### Data Collection and Data Analysis

Prior to the start of the intervention, a team of specialists along with the project staff introduced a new case sheet in all 8 facilities through a one-day on-site training. This team provided a brief refresher on skilled birth attendance as part of the orientation process and focused on the use of the case sheet as a job aid and for documentation of the delivery process. The case sheet had 2 components: (1) delivery record providing clinical information relevant to delivery care, and (2) complication case sheets providing information on diagnosis and management of maternal and newborn complications, specifically focused on PPH, PIH/eclampsia, and birth asphyxia. Photocopies of the completed case sheets from all 8 study facilities were sent monthly to the project office, which remained the primary source of data on maternal and newborn care practices. Independent obstetric and neonatal experts who were oriented about the study protocols reviewed these case sheets and marked the diagnosis (per study definition) and management (per national health mission protocols) of a complication (primary outcome) as correct, incorrect, or incomplete.

For the secondary outcome of provider knowledge, the providers completed a knowledge questionnaire before and after the intervention. The questionnaire consisted of 21 items on obstetric content (initial assessment and labor, PIH, and PPH) and 15 items on newborn content (infection control, neonatal resuscitation, routine care, and care of low birthweight babies). The maximum score that providers could obtain was 50 (29 points from the obstetric component and 21 points from the newborn component), and they had up to 30 minutes to complete each section. Specialists involved in the development of the training content validated the questionnaire. In the intervention facilities, all participants completed the pretest before the skills refresher session (n = 73). The post-intervention test was restricted to those who had taken the pretest and had attended one of the drills sessions (n = 50). In the comparison facilities, all nurse providers and medical doctors associated with maternal and newborn care (n = 36) took the knowledge tests at the beginning and at the end of the study.

To assess provider skills, we used an objective structured clinical examination (OSCE). For obstetric content, we had 6 observed (32 points) and 4 unobserved (18 points) stations focused on assessing the ability of the staff nurses to identify maternal complications and to begin initial management based on guidelines. For newborn skills, there were 5 observed (36 points) and 5 unobserved stations (14 points) that focused on assessment of a newborn, resuscitation steps, and care of a low birthweight baby. Participants were expected to complete the tasks in 4 minutes. A team of 4 assessors conducted the skills assessment: at the intervention facilities, 50 staff members who had attended the skills session and at least one drill session took the test; in the comparison facilities, 35 staff associated with labor and newborn care took the test. All assessors (obstetricians, pediatricians, and nurses) attended a standardization workshop and received a checklist so that observations were reliable.

At the end of the intervention period, a descriptive qualitative study involving in-depth interviews (one-on-one and group-based) with the health care providers was carried out at 3 intervention and 2 comparison facilities. The interviews helped understand the experiences of these providers, capture best practices, and highlight some of the main challenges with the intervention. The team of interviewers (4 nurses along with project staff) underwent a 2-day orientation and training process on qualitative methods of data collection and note taking. Teams of 2 interviewers plus 1 project staff member (who served as an observer and note taker) conducted in total 12 semi-structured interviews with 19 participants comprising medical doctors (one-on-one interviews) and staff nurses (group interviews). Informed written consent was obtained from all providers with clear information and instructions provided regarding the objectives and their rights of participation in the interviews. All interviews were recorded except interviews with 2 medical doctors who did not want to be recorded. Most interviews with the doctors were in English while those with the nurses were in the local language of Kannada.

All data from cases sheets were entered into a database using Epi Info (version 3.5.2, 2008). For evaluation purposes, data from November 2013 to October 2014 were analyzed. Descriptive statistics were reported using mean and standard deviation for all the continuous variables and number and percentages for categorical variables. Cross-tabulations were used to compare the categorical variables. For all outcomes of interest, numbers and percentages were compared between 2 groups using *z* test of proportion. A *P* value less than .05 was considered statistically significant. All the analyses were performed using SPSS 18.

Completed knowledge questionnaires were scored by project staff using an answer key, and completed OSCE sheets were collated and individual scores compiled. The individual knowledge scores and OSCE scores were then entered into Microsoft Excel.

**FIGURE 1.**
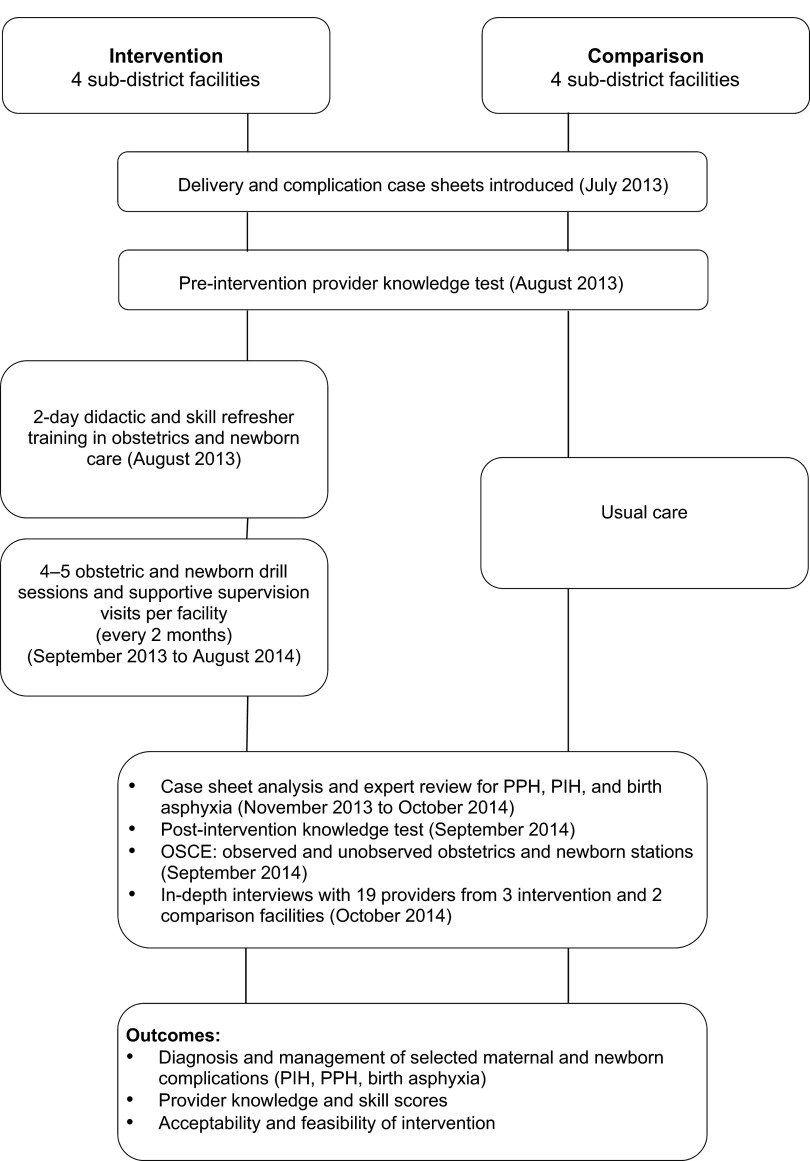
Flow Chart Depicting Study Design, Timeline, Tools, and Study Outcomes Abbreviations: OSCE, objective structured clinical examination; PIH, pregnancy-induced hypertension; PPH, postpartum hemorrhage.

The recordings of the interviews were translated and transcribed; the transcriptions along with the interview notes were analyzed to create topical summaries of the discussion with a special focus on the feasibility and acceptability of the intervention as well as documentation of any changes in obstetric and newborn care practices in the intervention facilities compared with comparison facilities. Figure 1 provides a summary of the overall study design and timeline.

Approval for this study was obtained from the Institutional Ethics Committee at St. Johns Medical College and from the Department of Health and Family Welfare, Karnataka, India. Consent was obtained from each facility in-charge prior to the start of the intervention at each facility.

## RESULTS

### Coverage and Use of Case Sheets

The large majority of providers in the intervention facilities attended the skills and drills sessions: 97% of the eligible doctors and nurses (9 doctors and 64 nurses) attended the skills refresher session, while 90% of them participated in at least one drill session. From November 2013 to October 2014, 6,452 deliveries were recorded in the 4 intervention facilities and 6,329 deliveries in the 4 comparison facilities. Spontaneous vaginal deliveries were significantly higher in the intervention facilities than in the comparison facilities (78% vs. 67%, respectively) (Table 2). No significant differences in the percentage of maternal or newborn complications were recorded—the percentages ranged around 1%–2% for PPH and birth asphyxia and 4%–5% for PIH. The rate of stillbirths was 9.6 per 1,000 births in the intervention facilities compared with 12.4 per 1,000 births in the comparison facilities (*P*=.87).

**TABLE 2.** Delivery Outcomes and Use of Delivery and Complication Case Sheets, Selected Districts of Karnataka, India, November 2013 to October 2014

Parameters	Intervention (N = 6452 deliveries)	Comparison (N = 6329 deliveries)	*P* Value
No. (%) of completed and analyzable records	5615 (87.0)	5204 (82.2)	<.05
No. (%) of deliveries			
Spontaneous vaginal	4369 (77.8)	3496 (67.2)	<.05
Ventouse/forceps	82 (1.5)	64 (1.2)	.86
Cesarean	423 (7.5)	621 (11.9)	<.05
No. of stillbirths (rate per 1,000 births)	62 (9.6)	79 (12.4)	.87
No. of fresh stillbirths (% of total stillbirths)	15 (24.2)	24 (30.4)	.52
No. (%) of complications recorded			
PIH	229 (3.5)	331 (5.2)	.35
PPH	69 (1.1)	41 (0.6)	.44
Birth asphyxia	107 (1.7)	104 (1.6)	.98
No. of complication case sheets completed (% of complications recorded)			
PIH	179 (78.2)	172 (52.0)	<.05
PPH	51 (73.9)	26 (63.4)	.56
Birth asphyxia	99 (92.5)	88 (84.6)	.09

Abbreviations: PIH, pregnancy-induced hypertension; PPH, postpartum hemorrhage.

Reported maternal and newborn complication rates were low in both intervention and comparison facilities.

Both intervention and comparison facilities used delivery records for almost all deliveries; however, 87% of records from intervention sites had analyzable data compared with 82% from comparison facilities (Table 2). The use of complication sheets to record diagnosis and management of complications was similar across intervention and comparison facilities, except for PIH cases (78% completed in intervention facilities vs. 52% in comparison facilities; *P*<.05). The case sheet analyses showed that the rate of recording of individual parameters was significantly higher in intervention facilities than comparison facilities for almost all maternal and newborn parameters (details available elsewhere).^14^

### Primary Outcome

There were no significant differences between intervention and comparison facilities in the diagnosis and management of any of the 3 primary complications, according to expert review of the complication sheets (Table 3). Among the 3 defined complications, correct diagnosis was lowest for hypertensive disorders of pregnancy (60% to 65% of cases diagnosed correctly in comparison and intervention facilities, respectively, compared with a range of 80% to 92% of PPH and birth asphyxia cases in intervention and comparison facilities). Correct management of complications was lowest for PIH cases (18% in intervention facilities and 20% in comparison facilities) compared with a range of 49% to 60% of PPH and birth asphyxia cases in intervention and comparison facilities.

**TABLE 3.** Diagnosis and Management of Maternal and Newborn Complications, Selected Districts of Karnataka, India, November 2013 to October 2014

Parameter	Intervention n/N (%)^a^	Comparison n/N (%)^a^	*P* Value
Correct diagnosis			
PIH	107/165 (64.8)	77/128 (60.2)	.43
PPH	37/42 (88.1)	23/25 (92.0)	.79
Birth asphyxia	72/90 (80.0)	63/72 (87.5)	.29
Correct diagnosis and management			
PIH	29/165 (17.6)	25/128 (19.5)	.79
PPH	25/42 (59.5)	13/25 (52.0)	.72
Birth asphyxia	52/90 (57.8)	35/72 (48.6)	.40

Abbreviations: PIH, pregnancy-induced hypertension; PPH, postpartum hemorrhage.

a Denominator is the total number of delivery records and complication sheets reviewed.

There were no significant differences between intervention and comparison facilities in the diagnosis and management of maternal and newborn complications.

Correct management of complications was lowest for pregnancy-induced hypertension, at around 20% of cases.

### Secondary Outcomes

**FIGURE 2.**
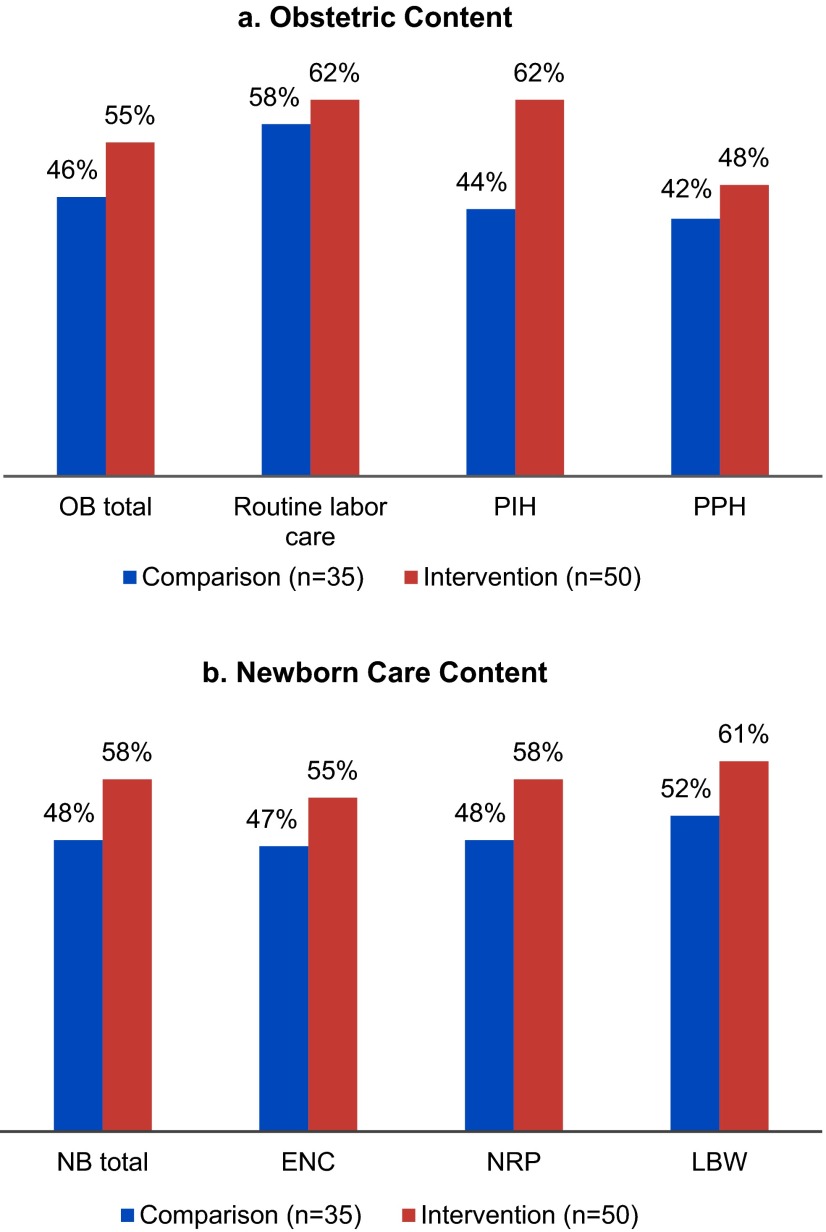
Post-Intervention OSCE Scores for Providers in Intervention and Comparison Sites by Obstetric and Newborn Content Abbreviations: ENC, essential newborn care; LBW, low birthweight; NB, newborn; NRP, neonatal resuscitation procedure; OB, obstetric; OSCE, objective structured clinical examination; PIH, pregnancy-induced hypertension; PPH, postpartum hemorrhage.

Average knowledge scores among providers were similar between intervention and comparison facilities both before and after intervention (Table 4). However, providers in the intervention facilities showed significant improvement between pre- and post-intervention in their average scores for both obstetric (49% to 57%; *P*=.006) and newborn care practices (48% to 56%; *P*=.03). No such significant changes in knowledge scores were noted among providers in the comparison facilities (Table 4). Providers in the intervention facilities scored significantly higher on the OSCEs than providers in the comparison facilities for both the obstetric skills (55% vs. 46%, respectively; *P*<.001) and newborn skills (58% vs. 48%, respectively; *P*<.001) (Table 4). Within obstetric skills, improvement in skills-related PIH management (62% vs. 44%; *P*<.001) contributed the most to this difference (Figure 2a). In terms of newborn care, all 3 components showed significant differences in scores between intervention and comparison facilities: essential newborn care (55% vs. 47%; *P*<.001); newborn resuscitation program (58% vs. 48%; *P*<.001); and care of low birthweight babies (61% vs.52%; *P<.001*) (Figure 2b).

**TABLE 4.** Maternal and Newborn Knowledge and Skills of Providers, Selected Districts of Karnataka, India, Before (August 2013) and After (September 2014) the Skills and Drills Intervention

Parameter	Intervention		Comparison		*P* Value
	Mean (SD)	%	Mean (SD)	%	
**Knowledge scores^a^**					
Pre-test	N = 73		N = 36		
Obstetric (maximum score = 29)	14.2 (4.3)	49	15.2 (3.6)	52	.21
Newborn (maximum score = 21)	10.2 (3.9)	48	11 (3.0)	52	.30
Post-test	N = 50		N = 36		
Obstetric (maximum score = 29)	16.4 (4.3)	57	16.6 (3.9)	57	.84
Newborn (maximum score = 21)	11.6 (3.3)	56	10.4 (3.3)	49	.09
**OSCE scores**					
Post-test	N = 50		N = 35		
Obstetric (maximum score = 50)	27.5 (4.9)	55	22.9 (5.8)	46	<.001
Newborn (maximum score = 50)	29 (5.2)	58	24.2 (6.2)	48	<.001

Abbreviations: OSCE, objective structured clinical examination; SD, standard deviation.

a There was a significant increase between pre- and post-test knowledge scores in the intervention group for both obstetric content (49% vs. 57%, respectively; *P*=.006) and newborn content (48% vs. 56%, respectively; *P*=.034). In contrast, there were no significant changes between pre- and post-test knowledge scores in the comparison group: obstetric content (52% vs. 57%, respectively; *P*=.12); newborn content (52% vs. 49%, respectively; *P*=.42).

Providers in the intervention facilities had significantly higher skill scores than in the comparison facilities.

During the in-depth interviews, the nursing staff indicated this intervention was acceptable and feasible, especially the drills component. They also reported on the usefulness of case sheets in improving documentation, although they all reported having had concerns initially with regard to workload. Almost all the respondents said that the case sheets had all the details required for assessment and management of the cases, which guided them further. Two of the senior staff nurses from a Taluk hospital said:

*Actually, it* [the case sheets] *has helped us in our work. We can be acquainted with what we are doing. ... We can decide what we should do further. ... So it guides us as to how to proceed in a case.*

With regard to skills refresher sessions, most of the nurses found the demonstration of the activities and practical sessions at the skills stations to be useful for refreshing their knowledge and skills.

*It* [the skills refresher sessions] *was useful because it refreshes our skills since we forget some of the skills. We can remember all such skills and apply* [them] *into our regular practice.*

The providers reported that drills training was very different from any other training they had received previously because the drills training focused on and improved their teamwork. Most of the nurses said that their teamwork and confidence level for managing complications had improved, which in turn reduced the need for referrals of some of the complications. They also highlighted that the drills prompted them to prepare emergency trays for PPH and PIH.

Providers reported that the drill exercises, which focused on improving teamwork, were very different from any other training they had received.

*Prior to the training we were not aware of effective teamwork. ... Some of us would have forgotten a few things, but yesterday* [during PPH case management drills] *as it was teamwork, even if one of us forgot something, others would remind them about those things.*

Medical doctors, administrators, and many of the nurses focused on some of the major health systems challenges that hindered their ability to function optimally, including shortage of trained staff, rotation of nurses across departments, and inconsistent availability of drugs and supplies.

## DISCUSSION

This proof-of-concept study showed that the skills and drills intervention resulted in some improvement in knowledge, skills, and confidence of providers (as depicted from knowledge and OSCE scores, as well as in-depth interviews). However, it was not adequate to result in significant improvements in diagnosis and management of maternal and newborn complications. It is, however, important to note that the level of documentation of maternal and newborn complications (as a percentage of total deliveries) was low and remained similar in both intervention and comparison facilities. The recorded rates of complications were 1% for PPH, 2% for birth asphyxia, and 4%–5% for PIH. This was much lower than the reported rates of 10%–15% from surveys done elsewhere in India.^15^ This may be due to a variety of reasons. For example, many of the nurse providers mentioned that they usually do not record complications that they manage successfully. In addition, they mentioned that with improved management of third stage of labor, PPH cases have dropped significantly. Other reasons may include the punitive nature of the health care system, which discourages staff members from documenting events accurately for fear of being penalized.

Although the skills and drills intervention results in some improvement in knowledge, skills, and confidence of providers, it was not sufficient to improve diagnosis and management of maternal and newborn complications.

On the other hand, the average knowledge and OSCE scores at around 50% to 60%, even after the intervention, and correct management of complications at around 20% are perhaps indicative of the need for a more comprehensive systems-wide approach, including but not limited to improved content, methods, and evaluation of both pre-service and in-service training of these providers. A recent study in Uganda showed that to improve nurses' documentation, apart from documentation redesign and continuous trainer support, broader changes were necessary including building a critical mass of competent staff, continuous education, and changes in nurse skill.^16^

Reports from studies that have evaluated training programs in India have emphasized the need for dedicated personnel and a trainee tracking system to ensure quality training management and implementation.^9^^,^^17^ They have also showed that strengthening essential supplies and supportive supervision is critical to practice and to retain the newly acquired skills. In other words, systems-level inadequacies related to human resources, governance, and supplies contribute to poor adherence to guidelines, which affects quality of care. Such issues were reported across various facilities in this study including unavailability of specialists, vacancies in key positions, rotation of nursing staff between departments, lack of clear accountability within the system due to inadequate supervision, and inconsistent availability of equipment and supplies.

### Limitations

Our study findings are limited by the following issues:

- A quasi-experimental evaluation design, in which intervention and evaluation occurred simultaneously during a 1-year time frame: Ideally, a longer intervention period followed by evaluation perhaps would have provided more clarity on the impact of such an intervention. However, as a proof of concept, the intervention was not designed for full-scale evaluation.
- Insufficient focus on the skills of providers: The state government preferred a short skills refresher session over a week-long training session. It is unclear, however, if a 1–2-day skills refresher session along with 4–5 visits by experts over the 1-year period was sufficient to bring about the minimum change in provider behavior necessary to have an impact on the defined outcomes.
- Inadequate monitoring system: The study could have benefited from a more robust monitoring and feedback system from trainers, so that issues with management of complications could have been identified during their visits.
- Other limitations related to study design include the absence of baseline OSCE scores, the inability to link knowledge scores with individuals, and the paucity of information on mortality outcomes. The focus of the study was on ensuring that the drills—a new concept for facilities—were planned and executed properly; thus, some of the monitoring aspects of the intervention were lacking.

## CONCLUSION

The skills and drills intervention designed as a proof-of-concept study was feasible and acceptable and had some positive impact on maternal and newborn knowledge and skills of providers. However, although all nurses reported a marked improvement in their capacity and confidence to handle complications, this did not translate to improved diagnosis and management of maternal and newborn complications. For long-term and sustainable improvements in quality of maternal and newborn care, changes in knowledge and skills of providers, although necessary, may not be sufficient unless combined with policies to address systems-level inadequacies such as those related to supportive supervision and availability of drugs and supplies.

## Notes

**Peer Reviewed**
